# MicroRNAs in thyroid function and pathology

**DOI:** 10.1186/1756-6614-6-S2-A7

**Published:** 2013-04-05

**Authors:** Joanna Bogusławska

**Affiliations:** 1 Department of Biochemistry and Molecular Biology, The Centre of Postgraduate Medical Education, Warsaw, Poland

## 

MicroRNAs (miRNAs) are short (19-25 nucleotides), non-coding RNA transcripts regulating gene expression by binding to their 3′ untranslated regions and causing inhibition of translation or mRNA cleavage. They appear to regulate multiple biological processes, including cell growth, differentiation and apoptosis. MicroRNAs are transcribed by RNA polymerase as primary longer transcripts, which are then processed by Dicer and Drosha rybonucleases into mature miRNAs.

The importance of miRNAs for normal function of the thyroid gland has been demonstrated in mouse models with inactivated DICER gene. This mutation resulted in development of thyroid gland with follicular disorganization which led to inhibition of thyroid hormone production probably through significant downregulation of PAX8, FOXE1, NIS and TPO expression. In consequence, severe hypothyroidism was gradually developed.

It was demonstrated that aberrant expression of miRNAs can be deregulated in different types of thyroid cancers, and could be responsible for tumour initiation and progression. Most studies have focused on analysis of miRNA expression in papillary thyroid carcinoma (PTC) and upregulation of several miRNAs (including: miR-21, miR-146a, miR-181a and miR-221) was observed. Further functional studies have determined the role of these molecules in PTC pathogenesis. It was shown that miR-221 and miR-222 negatively regulate p27Kip1 (regulator of cell cycle progression) as well as KIT gene (a tyrosine kinase receptor involved in cell growth and differentiation). Interestingly, our recent studies revealed that miR-21, miR-146a, miR-181a and miR-221 contain binding sites in 3’UTR of THRB gene, coding for thyroid hormone receptor β which is an important tumour suppressor. We have demonstrated that the expression of this miRNAs is regulated by thyroid hormone - triiodothyronine (T3). These results suggest a new feedback control mechanism within the thyroid hormone signaling pathway.

Recent studies confirmed negative regulation of miRNAs expression by T3 in liver of hypothyroid mouse compared with euthyroid animals. Significant overexpression of miR-206 and downregulation of its target genes in mouse with low thyroid hormone levels was observed.

In summary, microRNAs play key role in thyroid gland development, thyroid hormone synthesis and proper function of T3 signal path. Aberrant expression and function of these molecules leads to thyroid pathologies including cancers. Current research is focused on the potential applications of microRNAs as novel diagnostic markers and therapeutic targets in thyroid cancers.

